# Microstructure and Air Trace Defects of the Rapidly Solidified ZK60 Magnesium Alloy Ribbon

**DOI:** 10.3390/ma17010030

**Published:** 2023-12-20

**Authors:** Shuai Bao, Chao Yang, Zhenshuai Li, Peiran Ye, Yungui Chen

**Affiliations:** 1 Institute of New Energy and Low-Carbon Technology, Sichuan University, Chengdu 610207, China; 2020323010012@stu.scu.edu.cn (S.B.); 2019323010026@stu.scu.edu.cn (C.Y.); 2019323010025@stu.scu.edu.cn (Z.L.); 2019223019127@stu.scu.edu.cn (P.Y.); 2 School of Materials Science and Engineering, Sichuan University, Chengdu 610065, China; 3 Engineering Research Center of Alternative Energy Materials & Devices, Ministry of Education, Chengdu 610065, China

**Keywords:** rapid solidification, ZK60, air mark, nanoindentation

## Abstract

ZK60 alloy metal ribbons were prepared successfully in a carbon dioxide atmosphere by varying the speeds of melt spinning. The thin metal ribbon with different solidification speeds was prepared by controlling different rotation speeds, and the influence of solidification speed on the ZK60 ribbon was studied. The results show that the gas mark has a significant effect on the local structure of the ribbon. The gas mark’s proportional area of the ZK60 ribbon increases first and then decreases with the increase in roll speed, and the gas mark proportion area is the smallest at 17.6 m/s. With the increase in the solidification rate, the base texture of the ribbon is enhanced, and the proportion of columnar crystals in the ribbon gradually increases. At the rate of 17.6 m/s, columnar crystals run through the entire side of the ribbon, and uniformly distributed spherical–particle phases are found inside the grain. At the speed of 17.6 m/s, the mechanical properties of different areas of the ribbon are close and different from those of the other two speeds, and the performance of the quenching zone is better than that of the slow-cooling zone.

## 1. Introduction

Rapid solidification (RS) can improve some defects, such as coarse grain and severe segregation, in conventional casting alloys, and it can improve the comprehensive properties of all aspects of the alloys [1]. According to previous studies, rapid solidification can bring about a hyperfine microstructure [1], a growth mode transition [2], anomalous solubility [3], and the presence of metastable phases [4]. In order to obtain a high strength and high elongation for metal materials, the rapidly solidified metal powder or the breaking of the rapidly solidified metal thin ribbon into particles is usually consolidated via hot extrusion or a laser-based powder bed fusion [5,6,7]. Recently, rapid solidification methods containing chilling techniques (e.g., melt drag and melt spinning), atomization techniques (e.g., water, centrifugal atomization, and gas), etc., have been commonly used [8]. Melt spinning (MS) can make the liquid metal quickly form a thin ribbon, and its cooling speed can reach 10^6^ to 10^9^ K/s, which is widely used [1].

Magnesium is known as the green material of the 21st century and the lightest structural material [9]. Limitations include the formability and strength associated with the restricted plasticity of the hcp structure of magnesium at average temperatures and low corrosion resistance. Compared with conventional synthesis and processing methods, the rapid solidification of new alloy compositions provides possibilities to overcome such limitations. Many researchers have paid more attention to the RS magnesium alloy. Through the rapid solidification technology, magnesium alloys with predominant properties can be fabricated. However, only a little research on the ribbon has been conducted through melt spinning in Mg alloys. Melt spinning is one of the most common methods used to fabricate rapidly solidified Mg alloys in the form of ribbons [10,11]. On the other hand, it has already been shown that the melt structure has a strong effect on the microstructure and various properties of as-spun RS alloys [8,10,11,12,13,14,15]. The literature on MS processing has been limited to the microstructural observations of melt-spin ribbons or flakes [16,17]. There have been studies on Long-Period Stacking Ordered structures in Mg-based alloys containing Rare Earth and Transition Metal elements [18,19], including studies on complex Mg-based alloys with enhanced creep properties [20,21]. Wang et al. showed that the commercial AZ91 alloy undergoes a cellular/dendritic transition during the melt-spinning process [10].

Rapid solidification and subsequent consolidation processes can yield excellent properties for the alloy. Most of the research focuses on the high performance of the metal after consolidation [5,22,23,24]. Few experimental studies have focused on the effect of the solidification rate on the microstructure and properties of magnesium alloys. The melt-spinning cooling rate is proportional to the wheel speed [25]. This study investigated the influence of solidification speed and air marks on the ribbon’s surface morphology, internal structure, element distribution, and mechanical properties by controlling roller speeds.

## 2. Experimental Procedure

The widely applied ZK60 Mg-alloy (6.06Zn–0.61Zr-balance Mg (wt.%)) was used in this study, which could obtain representative results. A total of 200 g ZK60 was heated and melted in a resistance heating furnace under the protection of a 60:1 atmosphere of carbon dioxide and sulfur hexafluoride. The molten metal was heated to 780 °C, which was about 145 °C above the liquidus temperature, and it was made to flow into the molybdenum roller at a particular speed and diameter of 48 cm through the nozzle hole with a diameter of 1.2 mm under the pressure of 0.02 MPa. In each group of experiments, only the speed of the roller was changed without changing other conditions.

The microstructure of the ribbons was observed using an optical microscope (OM), scanning electron microscopy (SEM), electron backscattered diffraction (EBSD), and transmission electron microscopy (TEM). The ribbon was embedded with a conductive cold-setting resin through sandpaper to 2000 mesh, and it underwent mechanical polishing before OM, SEM, and EBSD. Before EBSD was performed, argon ion polishing of the sample was also required to remove the surface stress. Etching for the ribbons was performed using a solution of 10 g of tartaric acid in 100 mL of H_2_O [26]. The structure of the ribbons was checked through X-ray diffraction using Cu-K_α1_ radiation. A profilometer measured the ribbon contact surface’s gas trace. The mechanical properties of the ribbon were evaluated through nanoindentation using a Berkovich pyramid-shaped diamond tip [27]. Before indentation, the samples were polished to a mirror-like appearance using a diamond spray down to 0.5 μm diamond particles. The maximum force was set at 20 mN, and the target drift was 0.1 nms^−1^. We marked several points from the roll-contact side to the non-contact side. The hardness (H) was derived from the load–displacement curves at the beginning of the unloading segment using Oliver and Pharr’s method [28].

## 3. Result and Discussion

### 3.1. Solidification Rate Calculation

The average thickness of the ZK60 ribbons produced by 8.8 m/s, 13.2 m/s, and 17.6 m/s on the wheel was about 162.5 μm, 130.2 μm, and 75 μm, respectively. The relationship between ribbon thickness and solidification rate can be obtained from previous studies [29,30].

(1) $$\frac{\partial T}{\partial t}=\alpha \frac{\partial T^{2}}{\partial x^{2}}$$

(2) $$\alpha =\frac{k}{cp}$$

where *T*, *x*, *t*, *ρ*, *c*, and *k* are the temperature (K), the distance from the interface (m), the cooling time (s), the density (kg/m^3^), the specific heat capacity (J/(kg B K)), and the thermal conductivity (W/(m B K)), respectively.

The general solution of temperature distribution of melt-spun ribbon is expressed as Equation (3)

(3) $$T_{1}=T_{i}+(T_{i}-T_{10})erf(\frac{\chi }{2\sqrt{\alpha_{1}t}})$$

The cooling rate at the thickness χ is given by Equation (4)

(4) $${\left[\frac{\partial T_{1}}{\partial t}\right]}_{\chi }=-\frac{b_{2}(T_{20}-T_{10})\chi }{2(b_{1}+b_{2})t\sqrt{\pi \alpha_{1}t}}exp[-(\frac{\chi }{2\sqrt{\alpha_{1}t}}{)}^{2}]$$

The solidification time of melt-spinning ribbon can be obtained from Equation (5)

(5) $$t_{s}={\left\{\frac{\sqrt{\pi }\chi \rho_{1}[L+c_{1}(T_{10}-T_{s})]}{2b_{2}(T_{i}-T_{20})}\right\}}^{2}$$

According to the above formula and parameters in Table 1, it can be obtained that the solidification speed of the ribbon is about 3.6 × 10^5^ K/s, 5.6 × 10^5^ K/s and 1.7 × 10^6^ K/s corresponding to wheel speeds of 8.8 m/s, 13.2 m/s, and 17.6 m/s, respectively.

### 3.2. The Gas Trace Morphology of the Ribbon at the Different Speeds of the Roller

**Figure 1:**
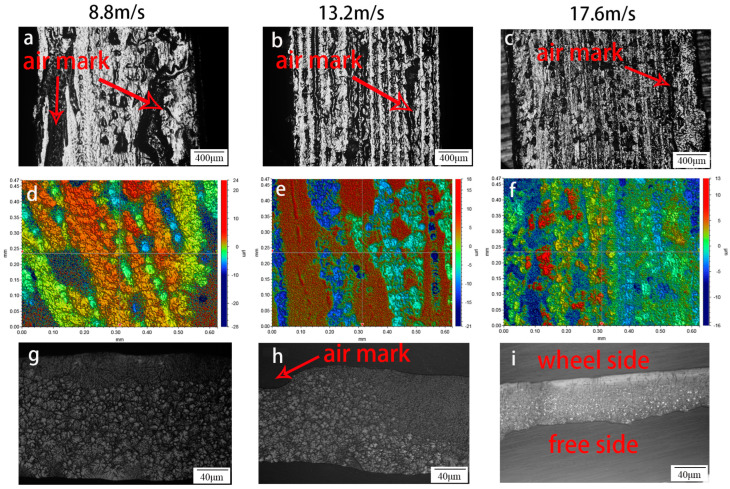
Optical micrograph of ZK60 ribbon at 8.8 m/s, 13.2 m/s, and 17.6 m/s, respectively (**a**−**c**), quench surface profile (**d**–**f**), and longitudinal section optical micrograph (**g**–**i**).

Figure 1d–f show the gas trace morphology between the molten alloy and the wheel observed by the profilometer. The gas trace morphology is mainly caused by gas between the molten alloy and the wheel during spinning [25]. Most “air pockets” appear in large lumpy strips along the direction of rotation. With increased rotational speed, the air marks gradually become continuous strips and then discontinuous small blocks. Other things being equal, this situation is mainly related to the metal solidification rate. According to the morphology meter result (shown in Figure 1d–f), the “air pocket” depth is the shallowest at the rolling speed of 17.6 m/s. The distance between the highest and lowest is 28.875 μm, and the Ra value of the 17.6 m/s ribbon is the lowest among the three speeds, indicating that the roughness of the ribbon at the rolling speed of 17.6 m/s is minimum. Due to the relatively lower cooling rate by carbon dioxide conductivity, the more significant the proportion of gas marks, the greater the influence on the solidification rate of the ribbon. Using the image Pro-Plus to calculate the “air pocket” area shows that the proportion of the “air pocket” area is 21.81%, 32.30%, and 15.64%, respectively, at the speed of 8.8 m/s, 13.2 m/s, 17.6 m/s. The test results of the profilometer can be obtained. The air mark is the deepest at 8.8 m/s and the shallowest at 17.6 m/s. As a result, the volume fraction of the “air pocket” in the ribbon is the smallest when the roll speed is 17.6 m/s among the three speeds. The main reason for this phenomenon is that when the roller speed increases, the alloy solidification speed increases, and the gas between the contact surface and the roller surface has no time to impact the alloy solution with a large area of gas marks. In addition to these reasons, the metal liquid is solidified and relatively dispersed. However, with the increase in the roller speed, the impact of the airflow on the metal liquid will increase, and the same effect applies to the solidification speed of the alloy. The actual number of “air pockets” is determined by the alloy solidification speed and airflow velocity. This phenomenon also appeared in Napolitano RE’s work [34]. Carbon dioxide conducts heat much less well than molybdenum. The existence of gas marks must significantly reduce the solidification rate of the region, resulting in the change in the microstructure and then affecting its mechanical properties. Among the three roll speeds, 17.6 m/s is suitable. From the longitudinal section shown in Figure 1g−i, the ribbons’ microstructure has apparent differences in different regions. It can be separated into two essential parts. One contacts the Mo roller (wheel surface) and the non-contact roller (free surface). The contact side is indicated in the figure, and this side consists of equiaxed and columnar grains. On the other hand, as we move to the other side of the image, well-defined grain growth is in the opposite direction of heat flux; see Figure 1g–i. Obviously, with the increase in roller speed, the thickness of the ribbon decreases, and the increase in cooling speed leads to the gradual increase in the thickness of the quench zone. The equiaxed and columnar crystal region is the cooling region, and the coarse equiaxed crystal region is the slow cooling region. Figure 1h remarkably confirms that the “air pocket” has a significant effect on the microstructure of the ribbon. Moreover, the change in the thickness of the quench zone around the air mark is also a good reaction that the existence of the air mark seriously affects the heat transfer in the melt-spinning process. At the speed of 17.6 m/s, the morphology of the ZK60 ribbon is a uniform distribution, which is similar to that in the rapid cooling zone of the other two groups.

### 3.3. Microstructure of ZK60 Alloy with the Different Speeds of the Molybdenum Roller

**Figure 2:**
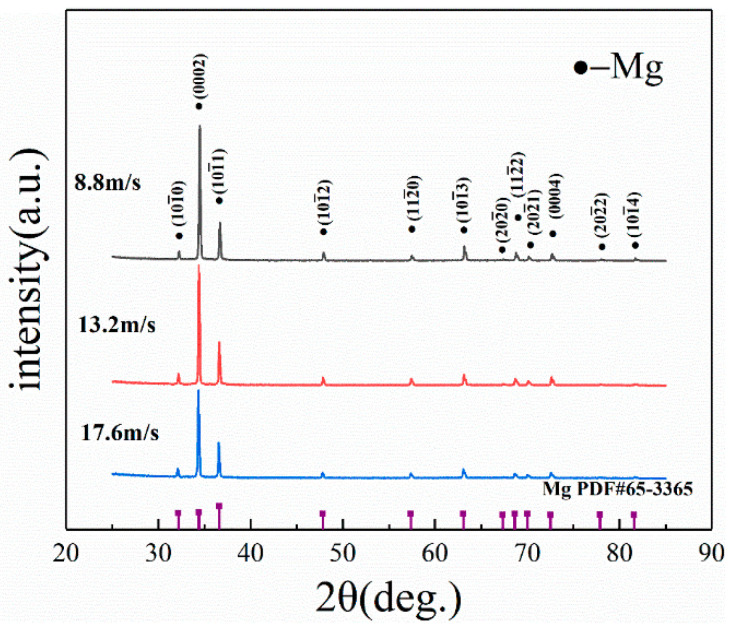
XRD patterns of the ribbon free side and wheel side at different speeds.

A comprehensive ribbons surface phase analysis was determined based on XRD patterns, as presented in Figure 2. As we can see, the phase composition of the three groups of ribbons is α-Mg without MgZn or MgZn_2_. The solid solubility is increased due to rapid solidification [35,36].

**Figure 3:**
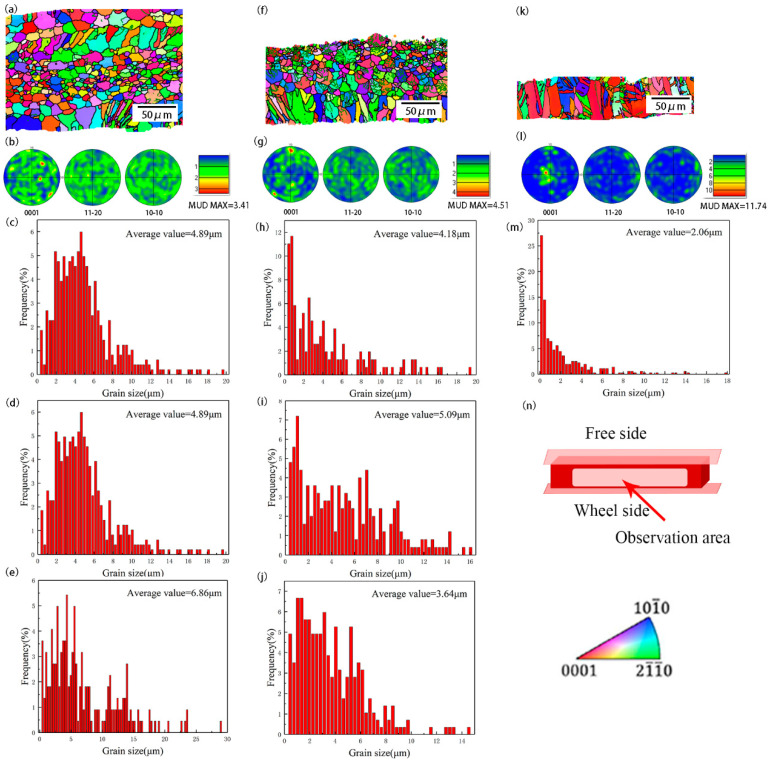
IPF and PF of the ZK60 ribbons manufactured at (**a**,**b**) 8.8 m/s, (**f**,**g**) 13.2 m/s, and (**k**,**l**) 17.6 m/s. Grain size distributions of the section (**c**,**h**) near the wheel side, (**d**,**i**) in the center and (**e**,**j**) near the free surface of the melt-spinning alloy ribbons with 8.8 m/s and 13.2 m/s, respectively. (**m**) The grain size distribution of the ribbon at a speed of 17.6 m/s. (**n**) Schematic diagram of beam direction.

Figure 3 shows the pole figure, inverse pole figure, and grain distribution in different ribbon regions. It can be seen from Figure 3a,f,k that with the increase in cooling rate, the proportion of columnar crystals in the ribbon gradually increases until columnar crystals penetrate through the entire ribbon. The increase in solidification speed leads to the rapid advance of the solid–liquid interface [37], and the solute has no time for segregation; there is no apparent constitutional subcooling area of the solid–liquid interface. As a result, the solid–liquid interface is pushed in the opposite direction of the heat flow relatively smoothly, resulting in the appearance of a large number of columnar crystals. Figure 3a shows that some columnar crystals appear on the cold surface, most of which are coarse equiaxed crystals, and very fine equiaxed grains appear in the middle part. When the solidification rate is not high, there is specific solute segregation. With the advance of the solid–liquid interface, the Zr element is pushed to the middle of the ribbon, and Zr has the role of refining magnesium alloy [38,39]. As a result, refined equiaxed grains appear in the middle of the ribbon. As the solidification rate increases, the apparent columnar and equiaxed regions appear in Figure 3f, and plentiful low-angle grain boundaries (LAGBs) appear in the equiaxial region. It is commonly believed that the formation of LAGBs is due to the gradual accumulation of a slight orientation deviation in the process of dendrite branching and growth [40]. Under stain-free conditions, the LAGBs need several nanoseconds to move [41]. Thus, the LAGBs→HAGBs do not have enough time to complete. There are more LAGBs in Figure 3f. With the increased rate of the solidification rate, the stability of the solid–liquid interface is improved, the growth trend of the crystals along the opposite direction of heat conduction is increased, and the proportion of the columnar crystal region increases. With the advance of columnar crystals, the solid–liquid interface gradually becomes unstable, and equiaxed grains appear. Moreover, due to the high solidification rate, equiaxed crystals are in a metastable state, and more small-angle grain boundaries appear inside [37]. As shown in Figure 3b,g,l, with the increase in solidification rate, the texture of the ribbon at (0001) is intensified, and many columnar crystals growing parallel to the direction of heat flow are formed in the strip with the increase in solidification rate. A characteristic of MS is the formation of pronounced growth textures. They can be explained by the preferential growth of favorably oriented nuclei. An (0001) orientation was observed for columnar growth in the magnesium alloys (e.g., Mg, Mg–Zn, Mg–Co) [42]. It can also be seen from the grain distribution diagram in Figure 3 that the grain in the ribbon presents an overall trend of decreasing with the increase in the solidification rate.

**Figure 4:**
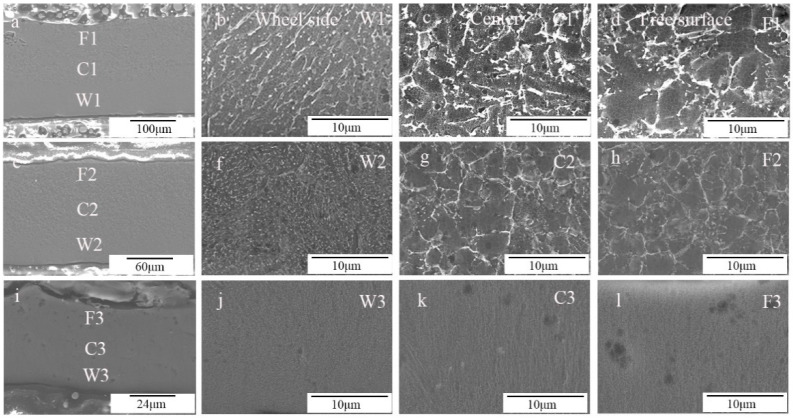
Cross-section SEM morphology of ZK60 ribbons manufactured at (**a**–**d**) 8.8 m/s, (**e**–**h**) 13.2 m/s, and (**i**–**l**) 17.6 m/s: (**a**,**e**,**i**) low magnification (×300) cross-section morphology, and high magnification (×5 k) morphology of the section (**b**,**f**,**j**) near the wheel side, (**c**,**g**,**k**) in the center and (**d**,**h**,**l**) near the free surface of the melt-spinning alloy ribbons.

We conducted SEM analysis of RS ZK60 alloy after etching. Figure 4a–l show the detailed cross-section SEM morphology of the RS ZK60 alloy at 8.8 m/s, 13.2 m/s, and 17.6 m/s, respectively. Figure 4b–d,f–h,j–l show the high magnification morphology of sections near the wheel side, the center, and the free surface of the ZK60 alloy ribbons, respectively. Well, with the increase in rotation speed, the microstructure of the ribbon has apparent changes, and the microstructure of different areas of the ribbon has a specific extent distinction at the same speed. According to Figure 4b–d,g,h, it can be seen that the size of the particles in the ribbon has a long strip-shape, and the particles near the wheel-side section become micro under the same condition. Figure 4f presents particles from clubbed to globular, which is smaller and more evenly distributed. As can be seen from Figure 4i–l, when MS ZK60 alloy is obtained at the speed of 17.6 m/s, there is almost no difference in the microstructure of the wheel-side part, middle region, and free surface region of the ribbon. The particles are small and uniformly spherical. Figure 4 further reflects that the microstructure of the ZK60 alloy ribbon is uniform at the roll speed of 17.6 m/s. These particles are not shown in EBSD, which may be produced after etching. After etching, the Mg matrix is corroded; there is specific element segregation between the matrix and grain boundary, some elements of the segregation together; at the same time, the solidification speed is fast, resulting in the element segregation being more dispersed, and no second phase is formed, so there is no appearance in EBSD.

**Figure 5:**
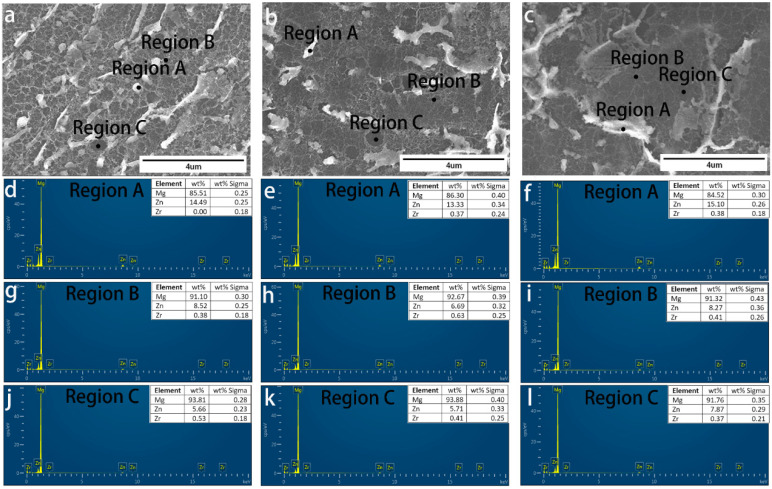
Cross-section SEM morphology of ZK60 ribbons manufactured at 8.8 m/s: high magnification (×13 k) cross-section morphology of section (**a**) near the wheel side, (**b**) in the center and (**c**) near the free surface of the melt-spinning alloy ribbons. EDS spectra of the particle (Region A in (**a**–**c**)) of sections (**d**–**f**), grain boundary (Region B in (**a**–**c**)) of sections (**g**–**i**), grain interior (Region C in (**a**–**c**)) of section (**j**–**l**), respectively.

**Figure 6:**
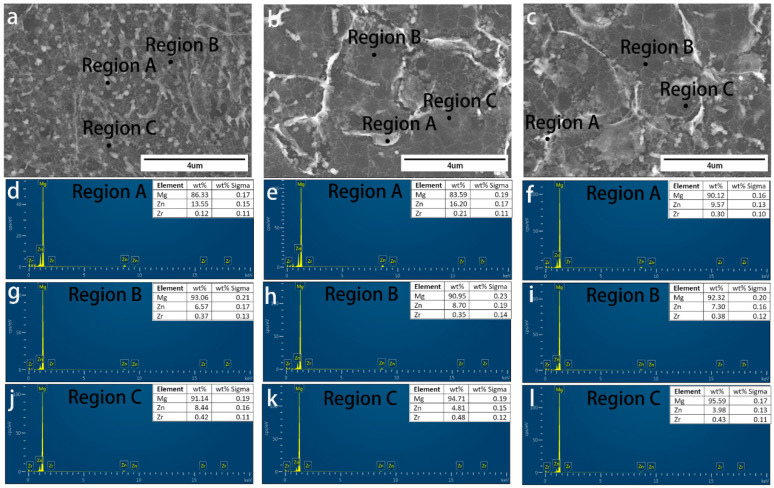
Cross-section SEM morphology of ZK60 ribbons manufactured at 13.2 m/s: high magnification (×13 k) cross-section morphology of section (**a**) near the wheel side, (**b**) in the center and (**c**) near the free surface of the melt-spinning alloy ribbons. EDS spectra of the particle (Region A in (**a**–**c**)) of sections (**d**–**f**), grain boundary (Region B in (**a**–**c**)) of sections (**g**–**i**), grain interior (Region C in (**a**–**c**)) of section (**j**–**l**), respectively.

**Figure 7:**
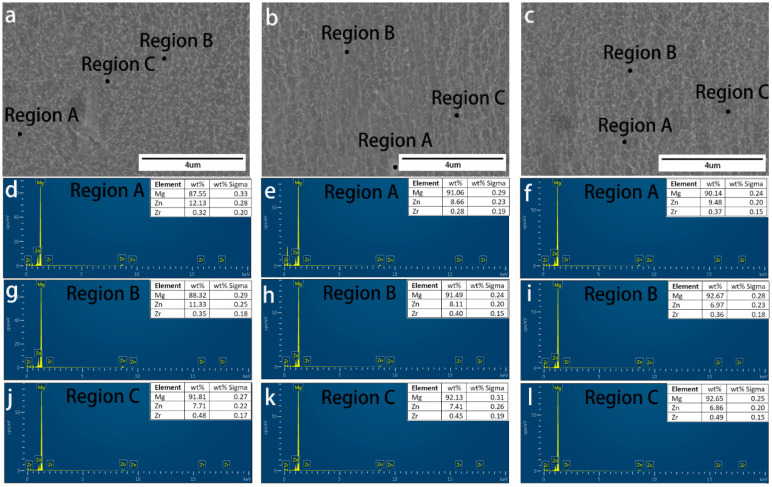
Cross-section SEM morphology of ZK60 ribbons manufactured at 17.6 m/s: high magnification (×13 k) cross-section morphology of section (**a**) near the wheel side (**b**) in the center and (**c**) near the free surface of the melt-spinning alloy ribbons. EDS spectra of the particle (Region A in (**a**–**c**)) of sections (**d**–**f**), grain boundary (Region B in (**a**–**c**)) of sections (**g**–**i**), grain interior (Region C in (**a**–**c**)) of section (**j**–**l**), respectively.

Figure 5, Figure 6 and Figure 7 demonstrate the SEM morphology and EDS spectra of the particle, grain boundary, and grain interior of the section near the wheel side, in the center, and near the free surface of ribbons manufactured at various speeds, confirming that the distribution of elements in different areas of the same ribbon is not uniform. The difference in EDS spectra also confirms the current organization’s heterogeneity, which is inconsistent with the uniform microstructure of the ribbon we expect. It is evident that the amount of elements dissolved in the crystal near the wheel side is higher than that in the other two areas; the different cooling rates in three areas of the ribbon lead to the change in solid solubility in the element. However, at the wheel speed of 17.6 m/s, it can be found that in different areas of the same region, the discrepancies shown by EDS spectra are less than those of the other two groups, and the material at the grain boundary is more nuanced and uniform, as illustrated in Figure 6. The grain distribution conforms to the normal distribution. Figure 7j–l show that at the velocity of 17.6 m/s, the grain size in the three areas of the ribbon has little change and tends to be almost the same corresponding to EDS and SEM results. In Figure 5, it can be found that there are more Zr elements in the middle of the ribbon, which corresponds to the results in Figure 3a. Zr refines the grain in this part.

**Figure 8:**
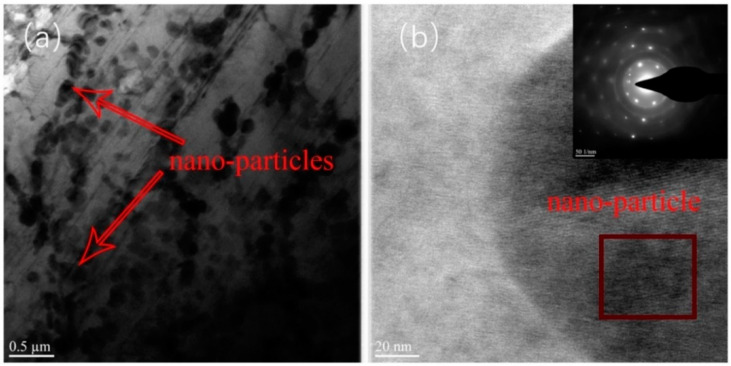
(**a**) Bright-field TEM image, (**b**) high-resolution TEM image and selected-area electron diffraction pattern of the particle in the ZK60 ribbon alloy produced at 17.6 m/s.

To further explore the particles in the ZK60 ribbon, a ZK60 ribbon of 17.6 m/s was selected for the TEM test, as shown in Figure 8. According to Figure 8a, it can be clearly found that there are a large number of 150 nm particles in the magnesium matrix, and there are quasicrystals in the rapidly solidified strip, which have been studied in many works of literature [43,44]. Figure 8b also shows that the black particles in the strip are quasicrystalline. Moreover, the corrosion resistance of the quasicrystals is high [45], so it confirms that the particles displayed on the surface of the ribbon after etching are quasicrystalline phase. With the increase in solidification rate, the quasicrystalline phase gradually nodules and distributes uniformly in the magnesium matrix.

### 3.4. Characterization of ZK60 Alloy with the Different Speeds of the Molybdenum Roller

**Figure 9:**
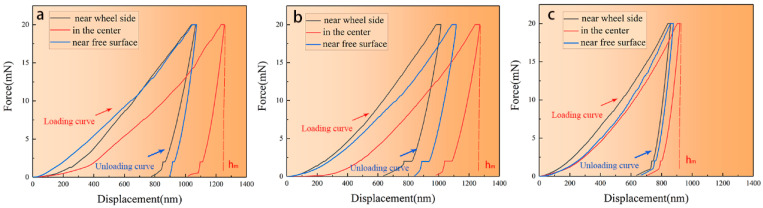
Representative load–displacement nanoindentation curves of the area near the wheel side, in the center, and near the free surface of the melt-spinning ZK60 ribbon manufactured at the speed of (**a**) 8.8 m/s, (**b**) 13.2 m/s and (**c**) 17.6 m/s.

In order to estimate the nanomechanical properties of the section near the wheel side, in the center, and near the free surface of the melt-spinning alloy ribbons, nanoindentation tests were performed to precisely measure the hardness values of all of the individual regions. The recorder force–displacement curve was calculated and analyzed using the Oliver and Pharr method [28]. Figure 9 shows the typical load–displacement nanoindentation curves of various areas of the melt-spinning ZK60 ribbon manufactured at different speeds. The peak load applied to ribbons was 20 mN. It can be clearly seen that the curves of the three regions have apparent differences in Figure 9a,b. On the contrary, the three curves are consistent in Figure 9c. The first stage was the loading stage before reaching the maximum indentation load. The elastic deformation gradually decreased, whereas the plastic deformation increased.

The second stage was the maintained loading stage. A short straight line occurred when the load reached the maximum value and stayed constant for 10 s. The third was the unloading stage. The indentation load was gradually reduced until it was completely unloaded. The indentation deformation consisted of elastic–plastic loading deformation and pure elastic unloading deformation. At the end of loading, the maximum depth reached by the indenter in different areas of the same ribbon is different, as shown in Figure 9a,b, which shows a difference in hardness among the section near the wheel side, in the center, and near the free surface of the melt-spinning alloy ribbons. It indicates the smaller the maximum depth, the greater the hardness. It can be seen from Table 2 that with the increase in wheel speed, the overall hardness of the ribbon increases. However, there is little change between 8.8 and 13.2 m/s. Because at 13.2 m/s, the air marks on the quenched-cold surface of the ribbon are the most serious among the three speeds, which leads to the reduction in cooling capacity and slowing down of the solidification rate. Although the speed is increased, the overall solidification rate does not change much. From Table 3, the performance of each area of the ribbon is very similar, considering the microstructure change, so we confirm that the ribbon at this speed is what we need.

Studies show that the average load is proportional to the yield strength (σ_y_) or compressive flow stress (σ_CY_) or maximum shear stress (τ_max_) of the tested material, so it can be known that nanoindentation hardness (H) is proportional to the yield strength (σ_y_): H ≈ C × σ_y_. Many studies show that for metal materials, when the yield stress is taken, the coefficient C ≈ 3 [46]. According to this, the yield strength of the three ribbons can be calculated; the highest and most consistent is the 17.4 m/s ribbon, which is about 384 MPa.

When the indenter presses into the material, the indenter does work, plastic and elastic work together when loading, and elastic works when unloading, by comparing the amount of plastic and elastic work to judge the plasticity of the material. In particular, the parts around the loading and unloading curves represent plastic work (Up), while the corresponding parts of the unloading curves represent elastic work (Ue). Equations (6) and (7) can represent Up and Ue, respectively [46]. Here, $f\left(x\right)$ is the loading section curve and $f_{1}\left(x\right)$ is the unloading section curve. By comparing the standard deviation (S1, S2) of the work completed by the plastic and the elastic, the uniformity of the ribbon itself can be judged, and the difference of mechanical properties of ribbon can be inferred. The calculation results are shown in Table 3. As the speed increases, the difference between elastic and plastic work is gradually reduced during the nanocompression process, further proving the ribbon’s uniformity.

(6) $$Up=\int_{0}^{h_{max}}f\left(x\right)-\int_{h_{r}}^{h_{max}}f_{1}\left(x\right)$$

(7) $$Ue=\int_{h_{r}}^{h_{max}}f_{1}(x)$$

## 4. Conclusions

In this study, the effect of solidification rates on the microstructure and properties of ZK60 during melt spinning was investigated as follows:

1. Under a carbon dioxide atmosphere, when the online speed is 17.6 m/s, the ZK60 ribbon gas trace ratio is at least 15.64%;
2. With the increase in solidification rate, the base texture of the ribbon is enhanced, and the proportion of columnar crystal in the ZK60 ribbon gradually increases until it penetrates through the ribbon;
3. A quasicrystalline phase appears in the rapidly solidified ZK60 ribbon. The 17.6 m/s ribbon quasicrystalline phase is spherical and evenly distributed in the magnesium matrix, and all parts of the strip have a uniform microstructure and mechanical properties.

## Figures and Tables

**Table 1:** Modeling parameters used to estimate the cooling rate [31,32,33].

Parament	ZK60	Mo
Initial temperature *T*_0_ (K)	1053 (*T*_10_)	300 (*T*_20_)
Thermal conductivity *k* (W/(m B K))	83.71 (*k*_1_)	147 (*k*_2_)
Specific heat capacity, *c* (J/(kg B K))	1360 (*c*_1_)	273.35 (*c*_2_)
Density, *ρ* (kg/m^3^)	1650 (*ρ*_1_)	10,200 (*ρ*_2_)
Latent heat L (kJ/kg)	290.93	

**Table 2:** Summary of the mechanical properties determined from nanoindentation, corresponding to the ZK60 ribbons manufactured at 8.8 m/s, 13.2 m/s, and 17.6 m/s: the section near the wheel side (W1, W2, W3), in the center (C1, C2, C3) and near the free surface (F1, F2, F3) of the melt-spinning alloy ribbons. Here, h_Max_ and H denote the maximum penetration depth underneath the indenter and hardness (see definitions in the text).

Sample	h_Max_ (nm)	H (GPa)
W1	1038.8	0.89
C1	1231.8	0.58
F1	1060.1	0.78
W2	1015	0.9
C2	1271	0.58
F2	1086.1	0.72
W3	856	1.23
C3	920	1.06
F3	876	1.17

**Table 3:** Summary of the plastic work and elastic work during nanoindentation, corresponding to the ZK60 ribbons manufactured at 8.8 m/s, 13.2 m/s, and 17.6 m/s: the section near the wheel side (W1, W2, W3), in the center (C1, C2, C3) and near the free surface (F1, F2, F3) of the melt-spinning alloy ribbons. Here, S1 and S2 denote the standard deviation of the plastic and elastic work (see definitions in the text).

Sample	Up (nm × mN)	S1	Ue (nm × mN)	S2
W1	6410.632173	561.4917	2339.953	377.8114
C1	7320.175548		1775.382	
F1	7758.867019		1422.623	
W2	6127.431315	299.612	2465.272	113.3588
C2	6424.778697		2284.148	
F2	6857.174349		2192.442	
W3	5710.625292	251.4887	1195.444	7.108149
C3	5491.70318		1198.968	
F3	5102.501641		1211.972	

## Data Availability

The data are included in the paper.
